# Ascitic Fluid Cytology Provides Diagnostic Clues in a Case of Gastric Metastasis from Invasive Lobular Carcinoma of the Breast

**DOI:** 10.70352/scrj.cr.25-0515

**Published:** 2026-03-06

**Authors:** Takashi Matsutani, Noriyuki Nishiwaki, Tomokazu Kakishita, Takaya Kobatake, Koji Ohta, Shinji Hato

**Affiliations:** Department of Gastroenterological Surgery, National Hospital Organization, Shikoku Cancer Center, Matsuyama, Ehime, Japan

**Keywords:** invasive lobular carcinoma, gastric metastasis, ascitic fluid cytology

## Abstract

**INTRODUCTION:**

Breast cancer metastasis to the gastrointestinal tract is rare, and invasive lobular carcinoma (ILC) shows a higher propensity for dissemination than invasive ductal carcinoma. Accurate diagnosis is often challenging because of nonspecific clinical and pathological findings.

**CASE PRESENTATION:**

A 53-year-old woman presented to our hospital with a diagnosis of gastric cancer discovered during gastrointestinal cancer screening. Fourteen years earlier, she had undergone a right mastectomy for ILC and subsequently received treatment for local recurrence and bone metastasis. The gastric lesion was initially diagnosed as primary gastric cancer, and adjuvant chemotherapy was administered after gastrectomy. However, postoperative ascites gradually worsened. Five years after gastrectomy, cytological examination of the ascites revealed tumor cells positive for GATA3 and estrogen receptor, and negative for E-cadherin, leading to a diagnosis of peritoneal metastasis from breast cancer.

ILC is characterized by a loss of E-cadherin and diffuse metastatic patterns, including peritoneal and gastric involvement. This case highlights the diagnostic difficulty of differentiating primary gastric cancer from metastatic breast cancer. Although tumor markers and imaging suggested gastric cancer recurrence, cytological examination revealed otherwise. Ascitic fluid cytology is essential for definitive diagnosis, emphasizing its diagnostic value for patients with ambiguous gastric lesions and a history of breast cancer.

**CONCLUSIONS:**

Metastasis should be considered in the differential diagnosis of patients with prior breast cancer presenting with gastric lesions. Ascitic fluid cytology can be a useful adjunct for an accurate diagnosis, guiding appropriate treatment, and avoiding unnecessary interventions.

## Abbreviations

CA19-9
: carbohydrate antigen 19-9
CEA
: carcinoembryonic antigen
EGFR
: epidermal growth factor receptor
ER
: estrogen receptor
FOLFOX
: folinic acid, fluorouracil, and oxaliplatin
HER2
: human epidermal growth factor receptor 2
IDC
: invasive ductal carcinoma
ILC
: invasive lobular carcinoma
PR
: progesterone receptor

## INTRODUCTION

Distant metastases of breast cancer typically involve the bones, lungs, and liver, and involvement of the gastrointestinal tract is uncommon.^1)^ ILC, a type of breast cancer, metastasizes more frequently to the internal genitalia, peritoneum, retroperitoneum, adrenal glands, bone marrow, and gastrointestinal tract than IDC.^2)^ Here, we report a case of gastric metastasis of ILC diagnosed by ascitic cytology 5 years after gastrectomy, which was initially diagnosed and treated as gastric cancer.

## CASE PRESENTATION

**Fig. 1:**
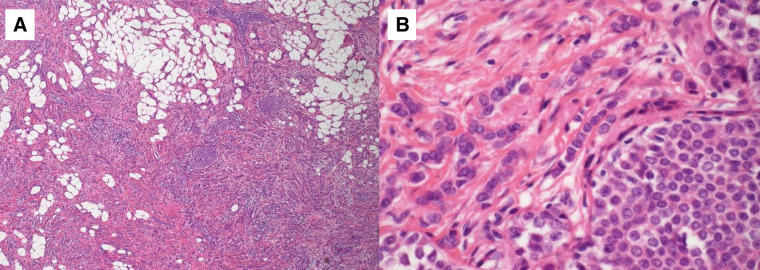
HE staining demonstrates discohesive invasive carcinoma cells infiltrating the stroma in a single-file pattern, a characteristic histological feature of invasive lobular carcinoma. (**A**) Lower and (**B**) higher magnification. HE, hematoxylin and eosin

**Fig. 2:**
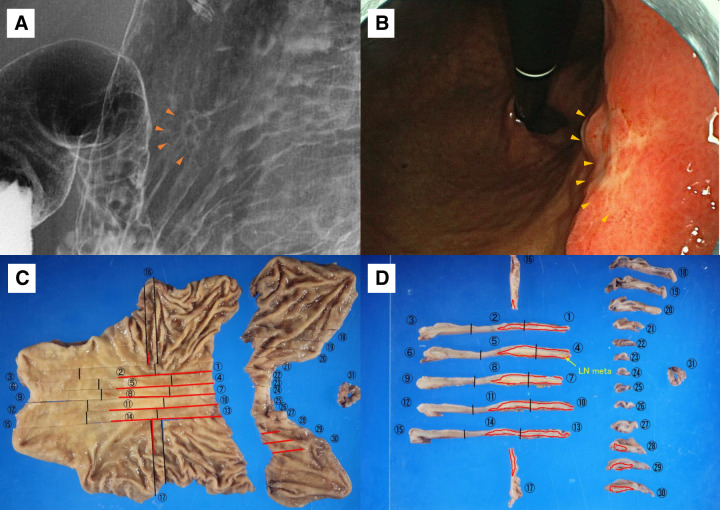
Preoperative imaging and pathological findings. (**A**) Preoperative upper gastrointestinal contrast image and (**B**) gastric endoscopy image. Orange arrowheads indicate depressed lesions present on the lesser curvature of the middle part of the gastric body. (**C**) The opened cut surface of the resected stomach, including the primary distal gastrectomy specimen and the additionally resected proximal stomach with the esophageal margin. The red lines indicate the location and extent of the tumor as well as the planes along which the specimen was sliced. (**D**) Serial cross-sectional slices were prepared along the lines indicated in panel C. Each slice is numbered sequentially. The red markings delineate the tumor area. LN meta is also shown. LN meta, lymph node metastasis

**Fig. 3:**
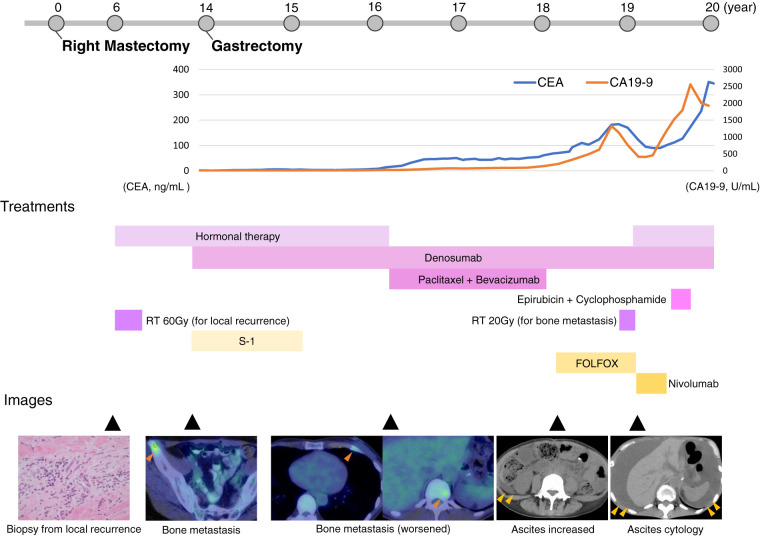
Clinical course with longitudinal changes in tumor markers, treatment timeline, and representative imaging findings at corresponding time points. Treatments targeting breast cancer are indicated in pink, while treatments targeting gastric cancer are indicated in yellow. The dark orange arrowheads indicate FDG accumulation in bone metastases on PET/CT. The light orange arrowheads indicate ascites on CT. CA19-9, carbohydrate antigen 19-9; CEA, carcinoembryonic antigen; FDG, fluorodeoxyglucose; FOLFOX, folinic acid, fluorouracil, and oxaliplatin; PET, positron emission tomography; RT, radiotherapy

**Fig. 4:**
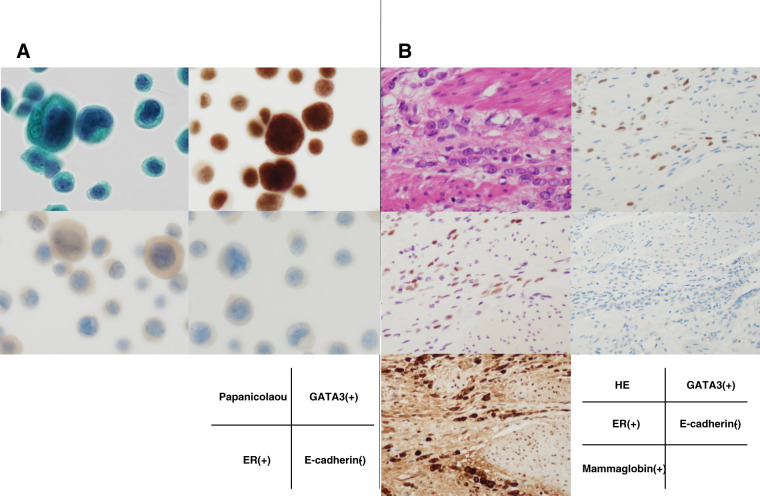
Microscopic findings of the ascitic fluid and the gastric tumor. (**A**) Cytological specimens from ascites. Papanicolaou staining shows clusters of atypical epithelial cells. Immunocytochemistry demonstrates positivity for GATA3 and ER, while E-cadherin is negative. (**B**) Histological findings of the gastric tumor. HE staining reveals infiltrating carcinoma with discohesive tumor cells. Immunohistochemistry shows positivity for GATA3, ER, and mammaglobin, with loss of E-cadherin expression. ER, estrogen receptor; HE, hematoxylin and eosin

A 53-year-old woman presented to our hospital with a diagnosis of gastric cancer discovered during gastrointestinal cancer screening. Fourteen years prior to the current presentation, she had undergone a right mastectomy for right breast cancer at our hospital. Pathological findings confirmed ILC, pathological T2N0M0 Stage IIA, ER-positive (ER+), PR- (PR+), and HER2-negative (HER2−) (**Fig. 1**). Postoperative treatment was not administered at the patient’s discretion. Six years after surgery, local recurrence occurred, and radiation and hormone therapies were initiated. Eight years after surgery, bone metastasis to the ilium was detected, and systemic treatment was continued at our hospital. At the time of her visit to our department, the local recurrence site had disappeared. Hormone therapy and denosumab were administered for iliac bone metastases that appeared 14 years after breast cancer surgery, and the disease was well controlled. Laboratory test results were normal. Upper gastrointestinal contrast (**Fig. 2A**) and gastric endoscopy (**Fig. 2B**) revealed a depressed lesion in the lesser curvature of the middle part of the gastric body. Endoscopic biopsy confirmed a diagnosis of poorly differentiated adenocarcinoma. CT showed no evidence of distant metastases. Following a multidisciplinary team meeting involving the attending physician (breast cancer specialist) and other specialists, primary gastric cancer was determined to be the most probable diagnosis, and treatment in our department was selected. Distal gastrectomy was planned based on the diagnosis of primary gastric cancer classified as clinical T1bN0M0, Stage IA. However, based on the pathological diagnosis during surgery, the surgical procedure was changed from distal gastrectomy to total gastrectomy owing to the presence of malignant cells in the proximal segment (**Fig. 2C** and **2D**). Intraoperative cytology was positive for malignant cells, and the patient was diagnosed with pathological T4aN2M1(CY1), Stage IV. S-1 was administered for 1 year as an adjuvant therapy for gastric cancer, and hormone therapy and denosumab for bone metastases from breast cancer were continued postoperatively. The bone metastasis had worsened 2 years after the gastrectomy, and the treatment regimen was changed to paclitaxel and bevacizumab. Four years after the gastrectomy, CT revealed increased ascites (**Fig. 3**), accompanied by elevated levels of the tumor markers CEA and CA19-9. Based on the diagnosis of recurrent peritoneal dissemination of gastric cancer, the chemotherapy regimen was changed from paclitaxel and bevacizumab to FOLFOX. After 2 months of administration, control of the ascites became poor, leading to a switch to nivolumab. Five years after the gastrectomy, the ascites became refractory to treatment, and cytology was performed. Cytological examination of the ascitic fluid showed that the atypical cells were GATA3-positive, ER+, and E-cadherin-negative, indicating peritoneal metastasis from breast cancer (**Fig. 4A**). A retrospective evaluation of the resected gastric specimen was performed, which showed the same immunostaining results as those of the ascitic fluid cytology and breast cancer specimen, indicating gastric metastasis and peritoneal dissemination from breast cancer (**Fig. 4B**). After diagnosis, although epirubicin and cyclophosphamide were administered, the treatment became difficult to continue within 14 weeks. She died 16 years after surgery for breast cancer and 6 years after gastrectomy.

## DISCUSSION

This report describes a rare case of metastatic breast cancer in which a patient with a history of ILC underwent gastrectomy for presumed primary gastric cancer, followed by adjuvant chemotherapy. During follow-up for increasing ascites thought to be of gastric origin, cytological analysis revealed breast cancer cells, leading to the re-evaluation of the gastric lesion, which was subsequently diagnosed as a metastasis from the original breast cancer.

ILC accounts for approximately 10%–15% of all invasive breast cancers and is considered a relatively uncommon histological subtype.^3–5)^ Although it is the second most common breast cancer subtype in the United States after IDC, its prevalence is lower in Japan, constituting approximately 5% of all breast cancers.^6,7)^ ILC is characterized by the loss of E-cadherin expression, leading to reduced cell adhesion and distinctive features such as single-file infiltration and diffuse growth patterns. Over 90% of ILC tumors are ER+ and/or PR+, and most are HER2−.^8)^ Biologically, ILC tends to have a low proliferative index (low S-phase fraction) and is often diploid, p53 wild-type, and EGFR-negative, reflecting a more indolent phenotype than IDC.^9–11)^

Although early-stage ILC shows a lower propensity for metastasis, advanced-stage disease often exhibits a unique dissemination pattern. Loss of cell adhesion due to E-cadherin deficiency contributes to transcoelomic spread rather than to hematogenous or lymphatic dissemination. Peritoneal carcinomatosis, gastrointestinal infiltration, and leptomeningeal spread are more frequently observed in the late stages of ILC. The most common metastatic sites include the peritoneum, liver, bowel, and ovaries, with the stomach being the most frequent site of gastrointestinal metastasis, followed by the small and large intestines.^9)^

Although breast cancer metastasis to the stomach is rare, a summary of 78 cases by Xu et al. suggested that gastrectomy may confer a survival benefit in selected patients with isolated gastric metastasis and well-controlled primary breast cancer.^12)^ Nonetheless, such cases represent systemic disease, and surgical intervention has not been shown to significantly impact overall survival. Systemic therapy remains the standard of care, while surgery is generally reserved for cases with acute complications such as gastric bleeding, obstruction, or perforation, to improve the QOL.^13,14)^

While reports have indicated that endoscopic biopsy and laparoscopic tissue sampling are effective for the diagnosis of gastric metastasis from breast cancer,^15,16)^ to our knowledge, this is the first report in which ascitic fluid cytology served as the basis for the definitive diagnosis of breast cancer metastasis. In retrospect, considering that gastric metastases from ILC are not exceptionally rare, this possibility should have been considered at the time of diagnosing the gastric lesion. Furthermore, since the ascites cytology was positive at the time of gastrectomy, immunohistochemical evaluation could have been performed at that stage. The delay in reaching a definitive diagnosis of peritoneal metastasis from breast cancer and the prolonged treatment course for presumed gastric cancer were partly due to the elevated levels of the tumor markers CEA and CA19-9, which suggested recurrent gastric cancer. However, these markers can also be elevated in cases of peritoneal metastasis from breast cancer,^17)^ and recurrence of breast cancer involving the peritoneum should have been considered. Elevated serum CEA and CA19-9 levels may reflect extensive peritoneal infiltration rather than tumor origin. Peritoneal dissemination or malignant ascites can increase the release and systemic absorption of tumor-associated antigens.^18,19)^ ILC has a characteristic metastatic pattern involving the gastrointestinal tract and peritoneum, and its diffuse growth pattern can clinically and pathologically mimic primary gastrointestinal malignancies.^2,20,21)^ This may partly explain why, although in a limited number of cases, treatment strategies for peritoneal carcinomatosis have shown temporary effectiveness in patients with peritoneal metastasis from ILC.^22)^ Therefore, reliance solely on serum tumor markers may be misleading, particularly in patients with a history of ILC. Metastatic breast cancer with peritoneal involvement should remain in the differential diagnosis, even when gastrointestinal tumor markers are elevated. Although uncommon, when patients with a history of breast cancer present with gastric lesions and a histological diagnosis is difficult, a comprehensive workup, including ascitic fluid cytology, should be considered to accurately determine the primary site and guide appropriate therapy.

## CONCLUSIONS

In patients with a history of breast cancer who develop gastric tumors, the possibility of metastatic disease should be carefully considered during pathological evaluation, and cytological analysis of ascitic fluid may serve as a valuable diagnostic adjunct.

## DECLARATIONS

### Availability of data and materials

The data supporting these conclusions are included in this article.
